# Would the Oceans Become Toxic to Humanity Due to Use and Mismanagement of Plastics?

**DOI:** 10.3390/ijerph22010017

**Published:** 2024-12-27

**Authors:** Jay N. Meegoda, William H. Pennock, Christina Brenckman, Ashish D. Borgaonkar

**Affiliations:** 1 Civil and Environmental Engineering, New Jersey Institute of Technology, University Heights, Newark, NJ 07102, USA; william.h.pennock@njit.edu (W.H.P.); cb433@njit.edu (C.B.); 2 School of Applied Engineering and Technology, New Jersey Institute of Technology, University Heights, Newark, NJ 07102, USA; ashish.borgaonkar@njit.edu

**Keywords:** plastics waste, microplastics (MPs), nanoplastics (NPs), ocean toxicity, corrective actions

## Abstract

The production of plastics and associated products, including microplastics (MPs), has been surging over the past several decades and now poses a grave environmental threat. This is because when not appropriately recycled, incinerated, or disposed of in fully contained landfills, plastic waste manifests as a potent pollutant, with vast amounts finding their way into oceans annually, adversely impacting marine life and ecosystems. Additionally, research also confirms there are direct impacts from MPs on water, air, and soil, impacting ecosystem and human health. This study investigated all aspects of plastics and microplastics such as their generation and consumption, their presence in oceans, and their ultimate fate. Next, a comprehensive literature search was performed to identify impacts MPs have on watercourses and soils and eventually on the ocean, taking into consideration the coupled impacts of metals and emerging contaminants adsorbed onto MPs. Then, a model to estimate the number of MPs in oceans and then using toxicity of MPs to humans and aquatic life to estimate when oceans would become toxic to humanity is described. Utilizing the model, it is possible to estimate the year when MPs in the ocean could potentially become broadly toxic, for both humanity and marine life, under different emissions scenarios. The estimates conclude that with the current MP discharge growth, oceans would become toxic to humanity between 2398 and 2456, for MP discharge growth only until 2020, it could be reached between 2408 and 2472, and for emissions ending in 2020, oceans would not become toxic to the humanity. Finally, remediation strategies are described to prevent oceans from becoming toxic to humanity by focusing on various action items such as education and awareness, reducing the utilization of single-use plastic, and conventional and innovative strategies that can be used for the treatment of stormwater and wastewater.

## 1. Introduction and Background

### 1.1. Plastic Generation and Consumption

The production of plastics has experienced a significant surge over the past seven decades. While plastic has undeniably conferred numerous benefits to modern society due to its cost effectiveness, versatility, and hygienic properties, the mismanagement of plastic waste poses a grave environmental threat. When not appropriately recycled, incinerated, or disposed of in fully contained landfills, plastic waste manifests as a potent pollutant, with vast amounts finding their way into oceans, adversely impacting marine life and ecosystems.

Plastic is progressively common due to its immense production rate and mismanagement of it as a material, specifically when it becomes a waste product. A comprehensive analysis conducted in a 2021 US congressional report identified the United States as the foremost contributor to global plastic waste, reflecting the ubiquity of single-use plastics in the American society [1]. MPs are endlessly produced from mismanaged plastic and are easily disseminated in the ecosystem due to their small size, light weight, and low density, making plastic management a global rather than local concern [2].

**Figure 1:**
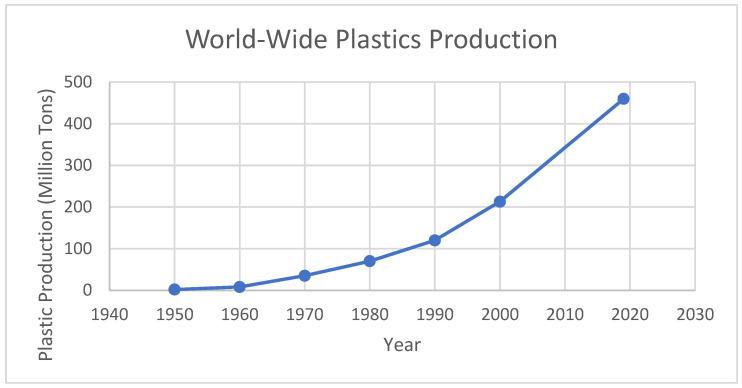
World-wide plastics production [3,5].

During the inception of the synthetic plastic era, global plastic production amounted to merely 2 million tons, which has since escalated to over 450 million tons, as shown in Figure 1 [3]. Additionally, roughly 56% of the total amount of plastics ever produced in the world were produced from the year 2000 up to the year 2020, highlighting the accelerating production of plastic [4].

The issue of plastic packaging exacerbates the problem, with a staggering 141 million tons out of 146 million tons of polymer plastics utilized for packaging being discarded in 2015 alone. Consequently, since 1950, cumulative plastic waste has amassed 6.3 billion tons, with a mere nine percent being recycled [3]. Alarming projections indicate that if current consumption patterns persist, landfills will house a staggering 12 billion tons of plastic waste by 2050 [3]. With plastic production expected to continue to grow, so will the amount of waste plastic generated. According to the latest forecasts by the OECD’s Global Plastics Outlook, within the next four decades it is projected that there will be 1014 million tons of plastic waste. Overall plastic waste is expected to triple by the year 2060 [5]. Although only a fraction of plastic waste ultimately reaches the ocean, most of it remains near coastal regions close to human populations and sensitive habitats [6].

The above trend is exacerbated by the rapid increase in plastic production over the past decade, surpassing the output of the entire previous century. Compounding the issue is the fact that virtually all plastic ever produced persists in the environment indefinitely, with estimates suggesting it takes between 100 to 1000+ years to degrade, but would remain in the ecosystem [7]. Presently, there exists an estimated 82–358 trillion pieces of plastic and MPs within the world’s oceans [8]. These materials aggregate into large concentrations within ocean gyres, forming what is commonly referred to as garbage patches, with the largest known example being the Great Pacific garbage patch located between Hawaii and California.

A study conducted in 2017 revealed that a staggering 50 to 100 percent of organisms inhabiting these deepest oceanic reaches had ingested plastic, including synthetic fibers such as Rayon commonly found in textiles. While certain plastics float, others, such as PET, possess a density greater than seawater, causing them to sink. Furthermore, through a process known as biofouling, floating plastic surfaces become encrusted with algae, barnacles, and various organisms, rendering them heavier and prone to sinking [6]. This expanding scope of research reveals the widespread presence of plastic pollution in places once thought to be pristine. Until recently, most studies on plastic waste have concentrated on the surface of the ocean. However, there is now a growing effort to explore deeper waters, sediments, freshwater sources, soil, air, and various biological systems for plastic debris. Without decisive action, projections suggest that by 2030, the mass of plastic in our oceans will surpass that of fish [4].

### 1.2. Where Do MPs End Up?

Secondary MPs, generated through the degradation of larger plastic items, and primary MPs undergo fragmentation into progressively smaller particles over time, eventually reaching nano plastic sizes (<1 μm) [9]. This perpetual breakdown process further perpetuates the dispersion of plastic pollutants throughout the marine environment, exacerbating ecological and health concerns.

Approximately half of the plastic waste generated finds its way into landfills [6]. Despite being engineered to contain waste, landfills often fail to confine plastic, particularly in regions with deficient waste management practices. The pervasive nature of plastic debris poses significant ecological risks, as evidenced by its propensity to escape landfill and waste dump sites, particularly in urban areas. Another common disposal method for plastic waste involves incineration. The incineration process releases carbon dioxide and other greenhouse gases, exacerbating the global climate crisis [10]. While advanced incineration facilities in affluent nations incorporate air pollutant filtration systems, many developing countries resort to open incineration practices, contributing 49 megatons per year of mass into the atmosphere [11].

**Figure 2:**
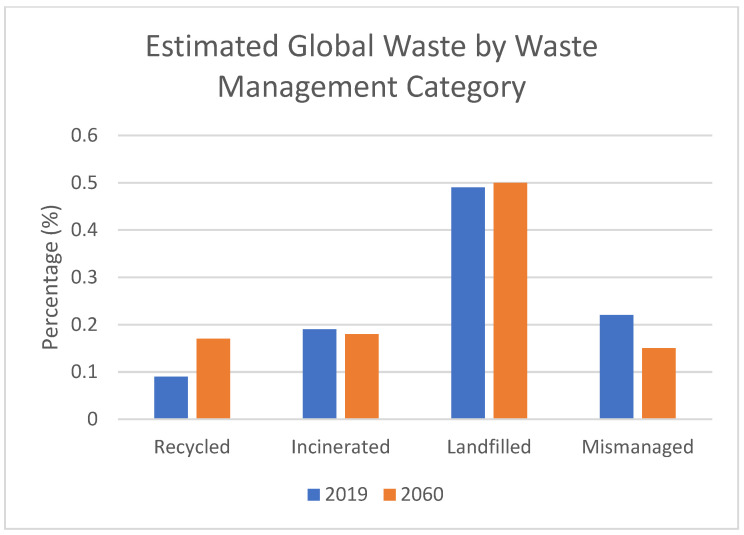
Comparison of global plastic waste based on waste management category between 2019 and 2060 [5].

In addition to improper waste management practices, plastic litter frequently infiltrates waterways during adverse weather conditions, such as heavy winds, rains, and storms. From there, it traverses via rivers and ultimately reaches the ocean. Depending on its buoyancy, plastic debris either sinks to the seabed, posing a threat to deep-sea organisms, or remains afloat for extended periods, eventually coalescing into immense aggregations of refuse, such as the notorious Great Pacific Garbage Patch [6]. Currently, only about 9% of plastic waste arrives at recycling centers [3]. These facilities process plastic waste into pellets and flakes, transforming it into viable feedstock for manufacturing purposes, conferring a renewed lease of life on discarded plastic and mitigating the environmental burden posed by its disposal. Figure 2 shows the total estimated global plastic waste found within the environment based on waste management category from 2019 to 2060. It is projected that during 2019–2060, recycled plastic will go from 9% to 17%, incinerated plastic will go from 19% to 18%, landfilled plastics will go from 49% to 50%, and mismanaged plastic will go down from 22% to 15% [5].

### 1.3. The Global Perspective on MPs

While Asia produces the majority of global plastic waste emissions into the ocean, wealthier regions are not absolved of responsibility. Despite contributing only around five percent of direct ocean plastic waste inputs, affluent nations often export substantial amounts of plastic waste to developing regions for processing, perpetuating the cycle of pollution [1].

When looking globally, many different countries have high levels of MPs in their waterways. A 2018 study revealed that 90% of 1393 ocean samples from various locations around the world contained MPs. The average concentration of MPs was 118 particles per liter, of which 91% were comprised of microfibers from plastic found in fabrics. The areas that contain the highest range of 151–250 plastic particles per liter were found in the Atlantic Ocean between North America and Africa [12].

The overall purpose of this study is to utilize the global ocean surface mass balance method put forward by Lebreton et al., 2019 [6] with the estimation of toxicity calculations to estimate the approximate year when oceans would become toxic at a broad ecosystem level. Methods to postpone this day will also be provided and referred to as remediation strategies. The study will utilize background information and the literature on MPs to express the impacts they have on various environmental aspects.

## 2. Literature Survey

MPs have acquired a reputation as contaminants that trigger concern in a vast number of environments including seas, soil, polar regions, rivers, and groundwater [13]. The most common forms of plastic measured include polypropylene (PP), polyethylene (PE), polyamide (PA), polyvinyl chloride (PVC), polystyrene (PS), and polyester (PES) and cellulose acetate (CA). As depicted in Table 1, the common forms of plastic are defined by their hydrophobicity/hydrophilicity and their density [14,15]. MPs also come in various sizes, from both the microscale and nanoscale, and can be found in the shape of microbeads, fibers, pieces, granules, pellets, and films [15,16,17]. MP particles contribute to distinct behaviors due to their characteristics such as hydrophobicity, high mobility, low density, and a large surface area-to-volume ratio [15].

The topic of MPs and the impacts they have on the environment is extensively researched. In fact, during 2010–2021, roughly 719 publications were published on the subject. Although, some progress has been made through this research, there is still a lot of different aspects of MPs that need to be studied [18].

### 2.1. Effects of Metals with MPs

It is important to point out that contaminants such as metals can be present in the environment and can be adsorbed onto MPs. This can have a direct effect on the organisms consuming the contaminated water and food sources. When metals are present in organisms, it is typical for the metals to accumulate in tissues, amplifying the risk of toxicity to the organisms [19,20]. Very limited research has been completed estimating MPs and metal levels in the tissues of wild fish and shellfish, and they have usually not shown any significant correlations [19,20,21,22,23,24,25,26,27,28,29]. These results may be due to both low exposure levels and the impact of abiotic and biotic factors, for example, water chemistries or species’ ecology [21,25,27].

Studies on both wild and farmed seabass revealed the occurrence of MPs as well as the trace presence of metals [21,22,24,25,26,27,28,29,30,31,32,33,34,35,36]. Although the amount of knowledge available is vast, no particular study has yet measured MPs and metals harbored in fish species. A study conducted by Abihssira-García et. al., 2020 confirmed the presence of MPs within the tissues of European seabass species located within a recirculation aquatic system (RAS) facility. A subsequent study emphasized the importance of water and feed as the primary pathways for MP exposure, which is a topic that requires further research [37]. They found that although occurrence of MPs was high (89% of specimens), the risk of metals exposure to human consumers was low [37].

### 2.2. Effects of Organic Contaminants with MPs

Organic pollutants, including pesticides, hydrocarbons, plasticizers, detergents, oils, and pharmaceuticals, have been observed to be bound to MPs within different environmental systems, specifically water bodies. Organic pollutants that lack hydrophilic functional groups may additionally occupy halogen replacement sites. These organic pollutants can potentially amass within food chains resulting in various ecological threats and adverse effects to species [32].

MPs can adsorb organic compounds that are present in the environment and can act as a pollution route of these chemicals for marine organisms. It is essential to assess the presence of these chemicals on MPs and their concentration levels because there is still uncertainty on the function of MPs in propagating persistent organic pollutants and biomagnification. Since identification and quantification of organic compounds adsorbed on MPs are lacking due to the homogenous sampling methods and standardized methodologies for the extraction, it is difficult to compare. When looking at collected data that are available for review, the most common organic pollutants correspond to phthalic acid esters and polycarbonates [31].

The sorption behavior can be affected by hydrophobicity and hydrophilicity, the surface charge, and the functional groups of the pollutants. Since hydrophobic affiliation can be a chief mechanism for sorption of MPs [34,38], organic contaminants with high hydrophobicity can be more readily absorbed on MPs [30,38]. Organic contaminants, particularly ionic compounds, can display diverse ionization states due to different aquatic conditions and therefore the sorption of organic contaminants onto MPs can be affected by electrostatic interaction. In regard to functional groups, the nitro group has the potential of attracting the π electron on MPs, resulting in the enhancement of the π-π electron–donor–acceptor interaction among the chemicals and MPs containing aromatic benzene rings [19,38].

## 3. Ways to Eliminate MPs in Oceans

The issue of plastic pollution in the oceans is a complex and growing environmental concern. Annually, it is estimated that between 4.8 and 12.7 million tons [25,39] of plastic waste are discharged from land into the ocean. A significant portion of this waste consists of synthetic polymers that are lighter than seawater, making up over 65.5% of global plastic production. Despite these substantial inputs, there appears to be a discrepancy in the expected versus observed amounts of floating plastic debris. Current estimates suggest there are more than 250,000 tons of plastic floating in the oceans, a figure significantly lower than the tens of millions of tons anticipated based on emission rates [6]. The degradation of plastics occurs in marine environments due to exposure to sunlight, physical wear, and oxidation which break down larger plastic items into MPs, particles smaller than 0.1 cm. However, the observed distribution of these MPs does not align with expected degradation patterns, suggesting that smaller particles are being removed from the surface water more quickly than anticipated. Potential pathways for this removal include ingestion by marine organisms, degradation into smaller (unmeasured) particles, incorporation into marine snow (aggregates of organic material that fall to the ocean floor), stranding on the ocean floor, and sinking due to biofouling, where organisms attach to the plastic, increasing its density [15].

The discrepancy between expected and observed amounts of floating plastic, along with the underrepresentation of MPs, highlights the dynamic and complex nature of marine plastic pollution. It underscores the need for improved waste management practices on land to prevent plastic from entering the oceans and for further research to better understand the fate of plastic once it reaches marine environments. Koelmans et al., 2017 [16] created a mass-balance-based model that considered emissions of macroplastics into the ocean surface from land and considered their removal by degradation into microplastics and removal by sinking out of the ocean surface. Lebreton et al., 2019 [6] created a detailed mass balance-based model similar to a Lagrangian dispersion model with geospatially distributed values of emissions, depths, currents, and wind. They added mechanisms of stranding at the shoreline, re-release of settled/stranded plastic back into the ocean surface, and transport of plastics from the continental shelf (depth < 200 m) to the offshore. Interestingly, when these three mechanisms, were neglected, Lebreton et al., 2019 [6] were able to reproduce the predictions of Koelmans et al., 2017 [16], who predicted that halting global emissions of macroplastics would lead to the removal of macro- and microplastics from the ocean surface within three years. However, Lebreton et al., 2019 [6] found that plastics in oceanic gyres with legible dates of production indicate that it takes 5 to 10 years for plastics to migrate from the coast to offshore gyres, which is incompatible with a period of three years for all plastics to disappear from the ocean surface. Their simulations showed that even if plastic discharge to ocean was halted in 2020, by 2050, the concentrations of macro- and microplastics would not even reach their levels in 2020. For microplastics, there would be a continuous increase through 2050 as macroplastics fragmented. This model and its findings suggest a more realistic outlook for the presence of microplastics in the ocean surface layer. Building on the findings of Lebreton et al., 2019 [6], the purpose of this research is to estimate the threat due to toxicity of the predicted persistence of plastics in the ocean surface. Hence, for the ease of comprehension, the details of the model proposed by Lebreton et. al., 2019 [6] is described below, including boundary conditions, data extraction, assumptions, and the outcome.

### 3.1. Model Overview

**Figure 3:**
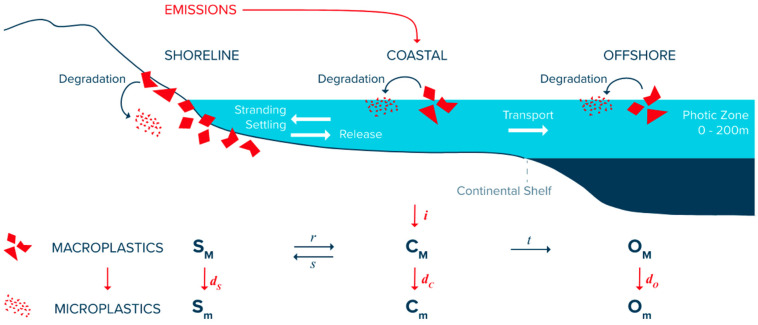
The estimated values of positively buoyant macroplastics in the ocean [6].

The study’s findings are concerning, as they suggest that even if no further plastic emissions occurred in the ocean after 2020, the level of MPs in the ocean could double by mid-century [9]. This is due to the slow degradation of already accumulated plastic waste into smaller pieces [32]. This is crucial as it highlights the need to take actions to remove plastic waste from the marine environment to prevent the generation of additional MPs in the decades to come. In order to understand the terms utilized in the mass budget of Lebreton et al., 2019 [6], it is helpful to view Figure 3.

The model proposed by Lebreton et al., 2019 [6] is a mass balance coupled with an ocean-scale transport model. The production of macroplastics for each individual year is input to calculate the global mass of plastic, D, produced within each year, $y_{0}$, and discarded in year y by utilizing the following equation:

(1) $$D\left(y,\;y_{0}\right)=\sum_{\sigma }^{Market\;Sectors}Production\left(y_{0}\right)\cdot Market\;Share\left(\sigma \right)\cdot Life\;Time\left(y-y_{0},\sigma \right),$$

where σ represents individual market sectors such as packaging or transportation, $Production\left(y_{0}\right)$ is the production of macroplastics for that sector in year $y_{0}$, $Market\;Share\left(y_{0}\right)$ is the percent of worldwide plastic production for market share σ, and $Life\;Time(y-y_{0},\sigma )$ is the probability density function developed by Geyer et al., 2017 [3] for plastic aging.

Buoyant plastic may strand back on the shoreline or sink from fouling-induced loss of buoyancy once at sea. At greater depths, debris may undergo rapid de-fouling due to microbial death in changing sunlight, oxygen, temperature, and salinity followed by resurfacing as they regain buoyancy. At shallower depths, debris has a higher chance of reaching the seabed. For this study, coastal waters are defined as the areas with bathymetry between the elevated tide line and the euphotic depth, which would be considered shallower than 200 m in depth. The model assumed that in the coastal environment, a proportion of floating plastic mass is caught by the landmass and undergoes repetitive episodes of stranding and release at the shoreline or settling and resurfacing from the seabed. A fraction of the amount of floating plastic that is still remaining at the surface may be transferred to offshore waters. After time progresses, the mass value of the stranded, settled, and floating plastic degrades into MPs, hence leaving the model domain. This model specifically concentrates on buoyant macroplastics and reflects that mass loss from degradation into MPs as a permanent sink [6].

Lebreton et al., 2019 [6] utilized the following equations to conserve mass. For any year between 1950 to the present, the accrued mass of plastic that has remained suspended in the oceans is equivalent to the summation of these 6 mass compartments: $C_{M},\;S_{M},$ and $O_{M}$, where C, S, and O refer to the coastal surface, shoreline, and offshore surface populations of plastic mass, respectively, and M and m subscripts refer to macroplastic and microplastic, respectively. For each year, a proportion i of macroplastic material discarded on land and reaching the costal surface layer $C_{M}\;$ was calculated. The release probability r that the material present in the coastal waters can strand or settle around shorelines $\left(S_{M}\right),$ and likewise, the material from the shorelines $\left(S_{M}\right)$ which can ultimately leak back into the coastal surface $\;(C_{M})$ was defined. The material from $C_{M}$ can escape the continental shelf and progress into the offshore surface layer $O_{M}$ with transport probability t. Lastly, coefficients $d_{S},\;d_{C},d_{O}\;$ represented the rate at which macroplastics that degrade into MPs, which are greater than 0.1 cm at the shoreline $S_{m}$, the coastal surface layer $C_{m}$ and the offshore surface layer $O_{m}$.

The mass budget was initiated with macroplastic input into coastal locations beginning in 1950 based on plastic consumption data. Then, from year y−1 to year y, Lebreton et al., 2019 [6] calculated the net mass input of plastic produced in year $y_{0}$, $\Delta (y,y_{0})$, into the surface waters of the global coastal environment by analyzing the following equation:

(2) $$\Delta \left(y,y_{0}\right)=i\cdot D\left(y,y_{0}\right)+r\left(1-d_{S}\right)S_{M}\left(y-1,y_{0}\right)+\left(1-d_{C}\right)C_{M}(y-1,y_{0})$$

where

y= year of input into the ocean;
$y_{0}=$ specific year of plastic production;
i= annual mass fraction of discarded plastic reaching costal ocean;
D= global plastic mass produced;
r= annual mass fraction of stranded or settled macroplastics that is released back into surface waters of coastal environments;
$d_{S}=$ annual mass fraction of settled or stranded macroplastics degrading into MPs;
$S_{M}=$ macroplastic material in the shoreline;
$d_{C}=$ annual mass fraction of floating macroplastics degrading into MPs;
$C_{M}=$ macroplastic material remaining from the previous year.

The mass balance of macroplastics is tracked in $C_{M}$, $S_{M}$, and $O_{M}$. For example, the concentration of macroplastics in the coastal surface was calculated as:

(3) $$C_{M}\left(y,y_{0}\right)=\left(1-s\right)\left(1-t\right)\Delta \left(y,y_{0}\right),$$

where

s= annual mass fraction of floating plastic that strands and settles around shorelines;
t= annual mass fraction of remaining floating plastic that is transported offshore.

The mass of macroplastics in the shoreline and the offshore surface was similarly calculated as $S_{M}$ and $O_{M}$. Of interest to the present study are the three population terms representing degradation into MPs from coastal, shoreline, and offshore environments. To illustrate, the mass of microplastics in the coastal environment was calculated as

(4) $$C_{m}\left(y,y_{0}\right)=\left(1+d_{C}\right)C_{M}\left(y-1,y_{0}\right)$$

The microplastic populations in the shoreline and offshore surface ($S_{m}$ and $O_{m}$) were calculated in the same way. Without detailed data on degradation rates, which were dependent on polymer, shape, and environment, Lebreton et al., 2019 [6] assumed that the degradation rates in all three environments were equivalent, although they noted that the shoreline environment would likely have a higher rate, $d_{S}$, than the constant d applied uniformly to the model and found it to be 3% by fitting.

(5) $$d=d_{C}=d_{S}=d_{O}$$

### 3.2. Model Assumptions

The degradation rate of macroplastics into secondary MPs is far more complex than described by this theory and the model. The values considered may be distinctively higher in terms of degradation rate for the environments considered to be along the shoreline when compared with surface waters. For example, microplastic particle shape and polymer type can frequently impact the values. Lebreton et al., 2019 [6] advised further research on degradation rates by both location and polymer types.

Another assumption within this study was that the model parameters do not exhibit any interannual irregularity and that the subtleties of degradation, stranding release, and recirculation into the coastal environment were independent of the age of plastic objects. Additionally, a fundamental limitation was the proportion of new plastic waste generation occurring on land that ultimately reaches the ocean, which was assumed to be a constant fraction of global plastic production from 1950 to the time of the study, which Lebreton et al., 2019 [6] estimated as i = 1.7–4.6%.

The model also does not explicitly consider processes that occur below the surface, including the biofouling and biofilm die-off that drives the settling and re-release of particles. The model assumed that the landmass was storing the majority of plastics. Lebreton et al., 2019 [6] noted that plastic debris buried under sediment layers could remain stored for an unknown duration and eventually be transported to greater water depths through sedimentary gravity flows.

### 3.3. Discussion and Interpretation of the Model Proposed by Lebreton et al.

**Figure 4:**
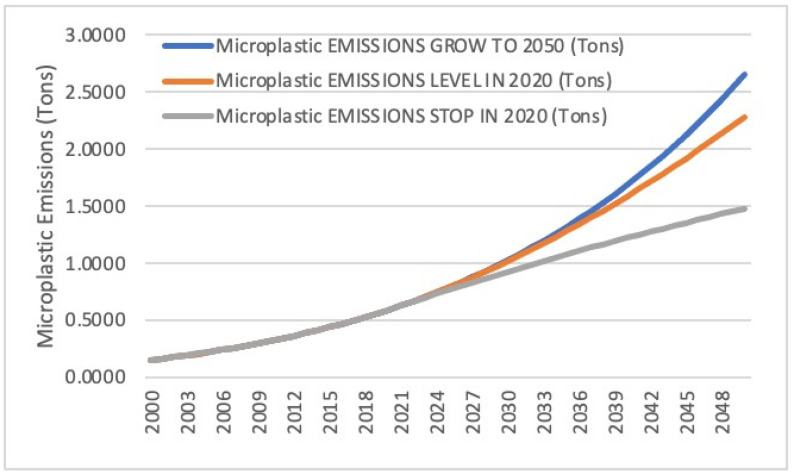
Total weight of MPs in the ocean if MP discharge grow at the current rate, level off, or stop from 2020 [6].

Lebreton et al., 2019 [6] made significant progress in understanding the amount of microplastics in the ocean. The prior Koelmans et al., 2017 [16] study had found that since 1950, roughly 99.8% of the plastic mass that entered the marine environment has degraded into micro- and nanoplastics that were then believed to have settled below the surface layer. As previously mentioned, Koelmans et al., 2017 [16] found that under a scenario with zero plastic emissions, almost all plastic would be removed from the ocean surface layer within three years, which Lebreton et al., 2019 [6] demonstrated to be implausible based on observed dates of production of sampled macroplastic. Using the previously described mass balance equation, Lebreton et al., 2019 [6] evaluated three scenarios moving forward from 2020: one in which the plastics discharge growth rate continue at that of the 2005–2015 period, one in which plastic discharges remained at 2020 levels indefinitely, and one in which all plastic emissions were halted in 2020 (see Figure 4). In the scenario where plastic emissions into oceans remain constant post-2020, the mass of buoyant macroplastics on the global ocean surface and coastlines continues to increase, although at a slower pace due to the degradation of older objects into smaller particles. The surprising result when comparing with Koelmans et al., 2017 [16] was that even where plastic emissions cease from 2020 onward, the mass of floating and stranded macroplastics decreases by 2050 to approximately 59% and 57% of their 2020 levels, respectively, rather than disappearing in three years as proposed by Koelmans et al., 2017 [16]. More concerning, the mass of MPs in the ocean and on the shoreline more than doubled from 2020 levels as remaining macroplastics in the environment gradually degrade.

Lebreton et al., 2019 [6] noted that their model does not account for the mechanisms of ingestion, aggregation, settling, stranding, and degradation into smaller particles for microplastics. Although their model does account for the degradation of macroplastics into microplastics in the ocean surface environment, it does not consider the direct discharge of primary and secondary microplastics into the ocean such as through rivers [40]. Lebreton et al., 2019 [6], as well as most surveys of ocean microplastics, do not consider particles smaller than 0.1 mm in diameter, which have been shown to be increasingly more toxic with decreasing size. Additionally, Lebreton et al., 2019 [6] found that while their model’s estimate of ocean surface microplastics for the year 2014 (0.28–0.75 million metric tons) was on the right order, measured values were about two-thirds lower (0.093–0.236 million metric tons). The correct order of magnitude, though, suggests that the work of Lebreton et al., 2019 [6] may be a good first-order approximation of the persistence of microplastics in the environment.

## 4. Plastic Toxicity

In their review of the environmental toxicity literature related to MPs, Weis and Alava, 2024 stated that “the chronic impacts and health risks due to the accelerated rate of plastic emissions and contamination of the global ocean environment and coastal zones are unprecedented”. Weis and Alava, 2024 also noted that there are major knowledge gaps in scientific understanding of the impact of microplastics, but that the weight of the evidence they reviewed supports a conclusion of causality of adverse effects by MPs. Further, they suggested that additional research is needed to obtain a more accurate assessment of exposure to microplastics and their potential impacts on human health. Hence, it is difficult to establish a toxic limit for microplastics, but an approximate value can be estimated based on limited evidence. Therefore, the following sections describe MPs’ effects on humans and MPs’ effects on marine life to establish an acceptable value for the toxicity of MPs.

### 4.1. Effects of MPS on Humans

**Figure 5:**
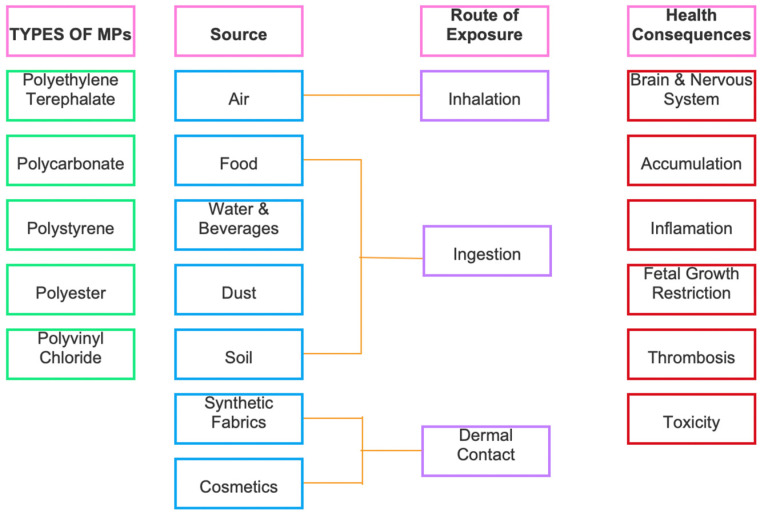
The various pathways humans are exposed to MPs.

Plastics have the potential to pose significant threats to both environmental and human health. Materials comprised of plastics typically contain dangerous carcinogenic properties that can affect the endocrine system of the human body, resulting in a variety of adverse health effects. These health effects encompass developmental, neurological, reproductive, and immune disorders. Additionally, plastics may introduce toxic contaminants which are directly transferred to marine life, and hence, consuming fish would eventually impact humans, resulting in further health effects. Figure 5 shows the different types of MPs, their source, route of interaction, and the health consequences for human exposure. There are three known exposure routes which include inhalation, ingestion, or dermal contact. Additionally, there are seven typical sources of exposure, including air, food, water, and beverages, dust, soil, synthetic fabrics, and cosmetics. The most common health consequences that are known to occur involve the brain and nervous system, accumulation, inflammation, fetal growth restriction (breastmilk transfer and placental transfer, thrombosis, and toxicity [41].

Various studies have shed light on the pervasive presence of MPs in the human diet, with estimations suggesting that an average individual may consume approximately 5 g of MPs per week, equivalent to the weight of a credit card [42]. These particles, sourced from diverse items, including seafood, sugar, salt, alcohol, bottled water, tap water, honey, and even air pose significant health concerns. Figure 5 shows various pathways in which humans are exposed to MPs.

Cox et. al., 2019 [43] also measured the values for the daily consumption of MPs based solely upon the gender and developmental stage of life of their participants. They concluded that adult males consume the highest with a value of 113 MPs per day. The age and gender category that attain the lowest daily consumption rate of MPs is female children with a value of 106 MPs per day [43].

Values for the approximate intake of MPs both annually and daily are shown in Table 2 for both consumption and inhalation. Utilizing data from Cox et al., 2019 [43], it was determined that roughly 363,416 MPs were consumed for the entire research population for both consumption and inhalation. For consumption alone, the total population had a daily value of 487 MPs, and an annual value of 177,655 MPs. In contrast, for inhalation, the total population has a daily value of 509 MPs and an annual value of 185,761 MPs [43].

### 4.2. MPs Effects on Marine Life

MPs can also have a detrimental effect on aquatic entities such as fish. Table 3 shows the primary effects MPs have on fish including their intestine, gonads, circulatory system, gills, olfactory sense, muscle, liver, immune system, and endocrine system [44]. However, the harmful effects on marine organisms are not limited to just fish. They can also affect crustaceans, mammals, mollusks, invertebrates, turtles, zooplankton, echinoderms, birds, phytoplankton, and bacteria. MPs have been discovered to cause adverse effects in mollusks. The effects include ingestion, accumulation, and bioaccumulation of MPs. These effects may lead to bodily impairment to the digestive tract, reduced feeding rates, changes in behavior, and toxicity [45]. The highest MP concentrations were found in big oysters, razor clams, and mussels [46]. The animals that are studied the most include fish, mollusks, zooplankton, crustaceans, and mammals [47].

### 4.3. A Toxicity Value for the Oceans

Annually, 1.13–2.24 million tons of plastic waste enters our oceans and ecosystems from US sources alone [1]. Once in the ocean, roughly 88% of the total is discovered floating close to the shoreline, 10% sinks to the seabed, and 2% is transported offshore on the surface [5]. While the mass of plastics floating on the ocean’s surface draws the most attention, it is only a fraction of the amount of plastic that actually enters the ocean [1]. With the staggering amounts of plastics being input into the oceans, it is crucial to determine a way to estimate the point when the level of toxicity will be too extreme.

Beiras and Schönemann, 2020 [48] projected that the maximum concentration for MPs below which large ecosystem effects are avoided should be approximately 13.8 mg/L for the larger MPs typically measured in oceanic surveys. This was estimated as the lower limit (5th percentile) of the toxicity threshold, which correlated with an experimental toxicity threshold of 44.7 mg/L for crustaceans (*Gammarus fossarum*) at which their growth was inhibited by MPs. However, this value is lower for nanoplastics since they are more dangerous, and research suggests 0.63, 0.006, and 0.002 mg/L as the safe concentrations for particle size ranges of 10–100, 1–10, and <1 μm, respectively, which likewise correlate to experimentally-derived toxicity thresholds for Korean rockfish and Pacific oysters [48]. Beiras and Schönemann, 2020 [48] also noted that preliminary data suggest that smaller MPs (10–100 μm) represent a fraction of a percent of the MP mass, leading them to focus on the threat of larger microplastics. It is important to note that dissolved oxygen can be diminished during consumption of MPs by zooplankton if their metabolism is not limited by iron. This is due to an alteration in zooplanktons’ diet that replaces their consumption of particle-bound carbon in part by MPs. The consumption of MPs by zooplankton reduces the grazing pressure on primary producers, resulting in an augmented export production and remineralization of organic particles leading to a loss of dissolved oxygen. There is a possibility that a direct effect of plastic pollution may have a negative impact on global oxygenation [49].

Using the above range of values of 13.8 mg/L to 44.7 mg/L for toxicity of microplastics and the prediction of the mass of microplastics in the oceans based on the model proposed by Lebreton et al., 2019 [6], one can predict the date when the ocean becomes toxic to the ecosystem. In order to accomplish the above, one must perform a curve fit of the three curves shown in Figure 4.

With reference to the model proposed by Lebreton et al., 2019 [6] with a plastics discharge growth rate continuing at the rate of the 2005–2015 period, MP mass in the ocean surface can be represented by an exponential curve shown below:

(6) $$Y=2.18\times 10^{-44}e^{0.0495x}$$

where x represents the year (e.g., 2020).

Likewise, by utilizing the model proposed by Lebreton et al., 2019 [6] with plastic discharges remaining at 2020 levels indefinitely, MP mass can be represented by an exponential curve shown below:

(7) $$Y=6.64\times 10^{-40}e^{0.0444x}$$

For stoppage of plastic discharge in 2020, which shows a leveling off, a logarithmic curve is more appropriate:

(8) Y=61.06ln⁡x−464.1

**Figure 6:**
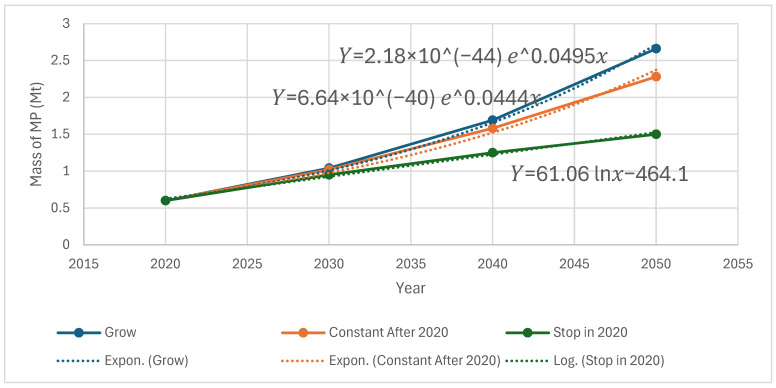
Total weight of MPs in the ocean if MP discharge grow at the current rate, level off, or stop from 2020 and fitted with trendlines [6].

Please note that all units of x are measured by year and units of Y are weights in tons. Figure 6 shows the model predictions as well as fitted curves. The next step would be the estimation of the mass of ocean water as shown below and then the mass of microplastic concentration corresponding to the range of values of 13.8 mg/L 44.7 mg/L for the toxicity of microplastics. Please note that the lower value of 13.8 mg/L for the toxicity of microplastics with respect to the definition of toxicity to ecosystems is to be taken as an order of magnitude. However, the combination of the findings of these studies represents a novel approach, and the authors wanted other researchers to consider this topic as it is an important topic for the survival of fragile ocean ecosystems and for humanity.

To obtain the concentration of MPs in the ocean, the total volume of the ocean in the world stays at 1.335819 × 10^9^ km^3^ [50], out of which the surface water occupies around 71% of the total volume [51].

Hence, the volume of sea water is 1.335819 × 10^9^ km^3^ or 1.33582 × 10^18^ m^3^. About 71 percent of the Earth’s surface is water covered, and the oceans hold about 96.5 percent of all Earth’s water [52], and hence, 68% of the volume of sea water or 907 × 10^15^ m^3^. Assuming the density of water, ρ, as 1025 kg/m^3^, the mass M of the surface water M = ρ × V can be estimated as M = 930 × 10^18^ kg. Now assuming the 13.8 mg/L or 11.04 ppm for toxicity of microplastics, a corresponding mass $M^{*}$ of microplastics in the oceans would be 10.3 × 10^12^ kg or 10.3 × 10^6^ Mt. For the higher limit of 44.7 mg/L or 35.8 ppm for the toxicity of microplastics, the corresponding mass $M^{*}$ of microplastics in the oceans would be 35.8 × 10^12^ kg or 35.8 × 10^6^ Mt.

Now, one can estimate the year to reach the above mass $M^{*}\;$ based on the model proposed by Lebreton et al., 2019 [6] and estimated from Equations (6)–(8). Hence, the year when MPs in the ocean could become toxic and reach the warning level for different emissions would be the following:

- By utilizing the model proposed by Lebreton et al., 2019 [6] and with the plastics discharge growth rate continuing at that of the 2005–2015 period (Equation (6)), the mass $M^{*}$ can be by either the year 2398 or 2456.
- Similarly, by utilizing the model proposed by Lebreton et al., 2019 [6] and with a plastics discharge rate in 2020 (Equation (7)), the mass $M^{*}$ can be reached by either the year 2408 or 2472.
- For plastic discharges stopping in 2020, the extrapolation extends to many millennia in the future, certainly beyond any validity. This indicates that the only safe strategy given the current knowledge is to attempt to completely eliminate plastic emissions into the ocean. This is especially worth considering, as the mass of small microplastics is unknown and is known to be far more toxic [48].
- The above analysis made many assumptions and should be only considered to warn humanity to take collective action to remediate the problem of microplastic contamination.

Please note that there were many assumptions and simplifications were made to obtain the above values, and hence, should only be used as qualitative values to highlight the importance of stopping the production as well as the use of plastics and also to develop appropriate policies and legislations to achieve the stopping of the production and use of plastics.

## 5. Remediation Strategies

The year 2398, when the ocean becomes toxic to the base of its food chain, should raise serious concern for the sustainability of life on earth, and humanity should take immediate and collective action as this suggests an upper limit of 374 years before accumulated ocean plastics become toxic. This may seem like ample time, but experience with climate change has shown how difficult it is to shift the course from an otherwise efficient technology when its use poses an existential threat. Hence, the best remediation strategy would be to stop the production of plastics which would yield the third scenario projected by Lebreton et al., 2019 [6] in which there is a chance for MP mass in the ocean to level off and eventually decline. It would, however, be politically suicidal for many nations, including developing nations, to stop the production and use of non-biodegradable plastics. Meegoda and Hettiarachchi, 2023 [53] stated that existing and growing abundance of MPs in the environment requires the use of multiple strategies to combat pollution. These strategies include reducing the base usage, public outreach to eliminate littering, and minimize single-use plastic use, reevaluation and use of new wastewater treatment, and sludge disposal methods, regulations on macro and MP sources, and a wide implementation of appropriate stormwater management practices such as filtration, bioretention, and engineered wetlands.

Hettiarachchi and Meegoda, 2023 [54] noted that combatting MP pollution requires preventive measures in addition to remediation. Although remediation is required, with the current technology, it may take a long time to be effective at scale. Prevention, on the other hand, can be and should be implemented immediately. The effectiveness of preventive measures works only if MP escape routes are studied and understood. Hettiarachchi and Meegoda, 2023 [54] also argued that such escape routes (rather loopholes) exist not only due to mismanaged plastic waste, but also due to cracks in the current waste management systems. One known MP loophole is from wastewater treatment plants (WWTPs). The inability of existing WWTPs to retain finer MPs in treated wastewater, which are finally treated wastewater from WWTPs, are released to water bodies such as rivers, lakes, and oceans, along with the return of captured larger MPs in bio-sludge back to landfills and their subsequent release into the environment through land applications, are a few examples. Other MP escape pathways include organic waste composting and upcycling of waste incineration ash. In addition, it is important to understand that the plastics that are in current circulation (active use as well as idling) are responsible for producing MPs through regular wear and tear. Closing these loopholes may be best attempted through policy interventions. Hence, humanity should consider a combination of following broader actions:

- Education and awareness;
- Reduce the utilization of single-use plastic;
- Conventional and innovative strategies for microplastic concentration, treatment, and destruction [53].

### 5.1. Education and Awareness

The first, and foremost strategy that needs to be implemented to remediate MPs is public education and awareness. The public will never understand the effects of MPs on the environment if they are not aware of the damage microplastics cause to their bodies and to the environment as well as the persistence and ubiquity of microplastics. People will not change behaviors that have a direct impact on MP accumulation if they are not aware and educated of the severity of the problem and the significance of their actions. In order to tackle the issues associated with MPs, and plastic pollution in general, not only is prevention needed, but also innovation. If society is educated and aware of the consequences of plastic pollution, there is a better chance of them altering their actions that contribute directly to plastic pollution as well as developing creative solutions to minimize their contribution to the problem. Plastic pollution and the impacts MPs have on the environment are not covered by the news or even taught in school systems. Therefore, it raises the question of how society will know and mount a response similar to the deterioration of the ozone layer by chlorofluorocarbons.

It has been posited that environmental education is one of the most imperative aspects in research about the socioeconomic influence on plastic pollution. A study disclosed that when there is just a 1% increase in environmental knowledge, there is a 0.4% increase in environmental behavior of university students [55,56]. According to Gifford and Nilsson, individuals with education and awareness of the subject are more concerned about environmental problems, which supports the hypothesized association between environmental awareness of individuals, and particularly, pro-environmental apprehension, behavior, and education [56]. Hammami et al., 2017 and Vicente-Molina et al., 2013 studied the factors affecting the pro-environmental behavior of students and found that pro-environmental behavior requires altruism and opportunity to practice. Although the strategies and the outcomes will vary based on the context, the pro-environmental behavior of the community will be amplified through an educational agenda, and the alterations of plastic pollution over time may be correlated to the educational efforts of a country [56,57].

The education of environmental aspects, particularly education of nature-based environmental aspects related to ecosystem preservation in primary and secondary schools, would be the first step in solving the planet’s environmental problems, which in return, provides a promising solution to mitigate plastic pollution among young people [27,53,54,56,57,58,59,60,61,62,63,64,65,66]. It is hypothesized that environmental education programs and campaigns will inspire students to investigate the plastic waste stream in daily life, resulting in a promotion of more responsible behaviors of young citizens. Respectively, environmental awareness and behavior rather than comprehension alone are integrated through curriculum improvement strategies by educators, which will steer the culture toward a lower plastic emission scenario with less costs than purely technological approaches [56,62].

In order to tackle the crisis of microplastic pollution, education and awareness on the topic need to be promoted. All aspects of MP issues need to be communicated to the public, including their numerous origins, types, effects, fates, and other related dynamics. To begin this process, these topics should be covered within school and university curricula. By initiating this topic early on, students and young people can become acquainted with the concern as early as possible [56,66].

The media has begun to raise public awareness of MPs in many countries. The British Broadcasting Corporation (BBC), has created various documentaries and television programs that present the issue of plastic pollution in a credible and easily understandable manner, resulting in the inspiration of the public to avoid using single-use plastic items. With these efforts, the media has contributed to enlightening people about the influence of MPs on the environment and encouraged them to curtail their use of plastics [56].

Presently, public awareness of the negative effects of MPs, particularly concerning health impacts, is either inadequate or non-existent. Therefore, it is crucial to urgently provide education and awareness to society of the damaging influence MPs have on humans and the environment. If public awareness increases, there is potential for an inclination to demand new policies to alleviate MP exposure. When these policies are applied, it is necessary that the research community conducts supplementary research to advance scientific knowledge and technology on the subject. In the long run, the alleviation of the burden of MPs on the marine environment will require the engagement of the general public, regulators, educators, and medical professionals. Table 4 shows the public outreach and community actions that could dramatically reduce the number of MPs entering both the environment and human body.

### 5.2. Reduce Use of Single-Use Plastic

The next action would be to reduce the use of single-use plastic on a daily basis. It is absurd to continue one-time use of plastic and discarding it when there are alternatives that can be used that will not increase the number of MPs present within the environment. Single-use plastics are an imperative example of the issues associated with the throwaway philosophy. Instead of investing in quality goods that will last, markets have promoted quantity and convenience over resilience. The global dependence on these plastics means accrual of waste at an astonishing speed. In fact, according to the United Nations Environment Program 2021, 100 million tons of single-use plastic is produced world-wide, one-third of total production. More alarming, one study found that approximately 90% of macroplastics found in the ocean were single use [67]. It is said that approximately 23 billion plastic bags in New York city alone are utilized by occupants on a yearly basis. Therefore, banning single-use plastic will not only diminish pollution, but also decreases the need for plastic production [53].

There are some techniques for encouraging the reduction in the use of single-use plastics, such as employing durable fabric bags for shopping excursions, opting for paper bags over plastic counterparts when making purchases, patronizing zero-waste grocery outlets and environmentally mindful retailers, substituting disposable plastic straws with reusable alternatives crafted from metal, glass, or bamboo, and curtailing reliance on single-use plastic water bottles by embracing refillable receptacles for hydration purposes.

Currently, there are more than 500 citywide ordinances banning single use plastic bags in the US, as well as 12 state-wide bans, which include California, Colorado, Connecticut, Delaware, Hawaii, Maine, New Jersey, New York, Oregon, Rhode Island, Vermont, and Washington [53]. The impact of such bans has been said to be the greatest in the state of New Jersey, where 5.5 billion single-use plastic bags were removed annually. Other jurisdictions have removed 45–200 million single-use plastic bags a year reliant on population size [68].

Aside from the ban of plastic bags, there have been bans on plastic foam in 11 states and more than 250 cities and counties, including Delaware, California, Colorado, Maine, Maryland, New Jersey, New York, Oregon, Vermont, Virginia, Washington, and the District of Columbia. It is evident that some of these states included do not have any bans on plastic bags, such as Maryland, Virginia, and the District of Columbia. There are also states that have banned plastic bags but not plastic foam, such as Connecticut, Hawaii, Rhode Island, and Washington [62].

The United States is not the only country acting. At least 20 different countries are taking some form of action to reduce their use of plastic and increase their recycling rates. However, please note that if care is not taken, the recycling of plastics would have the effect of releasing large quantities of microplastics into the environment via reprocessing. This is probably one of the most important sources, along with the fragmentation of plastic in the oceans. These emissions and many others could easily be reduced by filtering air and especially wastewater.

### 5.3. Conventional and Innovative Strategies for Microplastic Treatment and Destruction

It is important to use the knowledge of science and technology to implement strategies such as conventional and innovative techniques to treat or destroy microplastics. Recently, several strategies have been developed to remove microplastics from the ocean. Physical collection is an essential step, as having a critical concentration of MPs is essential for physical, chemical, and biological destruction methods to be efficient. Kibria et al., 2023 noted that large-scale transport in the open ocean is a very efficient way to transport MP debris long distances. Although these tend to accumulate in gyres far from land, several innovative techniques to remotely collect these materials, including the sea bin and the ocean clean-up array within the marine environment as well as interceptors in rivers upstream, take advantage of long-range transport of MPs to collect and treat them [69]. One promising approach to these collected debris is to recycle them, convert them to fuel, or incinerate them for energy [69].

The primary focus of management tactics for MPs is their abstraction from aquatic ecosystems. Of these techniques, there are two broad categories which include conventional strategies and advanced strategies. Conventional strategies include mechanisms such as coagulation, membrane bioreactor technology, rapid sand filtration, and adsorption. On the other hand, advanced techniques include electrocoagulation, photocatalytic degradation, electrochemical oxidation, and magnetic separation [63]. Fish et al., 2023 described the state-of-the-art physical, chemical, and biological methods for removing and destroying new and legacy microplastics in aquatic ecosystems (natural and urban). Fish et al., 2023 also stated that currently, there are no standardized, accepted, and cost-effective methods for the complete removal of microplastics from aquatic ecosystems [70]. They showed gaps in knowledge and recommendations for future research to help inform practice and legislation. A key consideration highlighted in the review by Fish et al., 2023 is that microplastics cycle through ecosystems—natural and engineered. These do not operate in silos, and waste from treatment processes could be a conduit for (unintended) the recontamination of microplastics. Hence, there is a need to take a whole-system-wide approach when developing innovative removal or destructive solutions, and ultimately, reducing plastic use remains the best option to safeguard future environmental and public health.

### 5.4. Advanced Technologies for Plastic Waste Disposal

**Figure 7:**
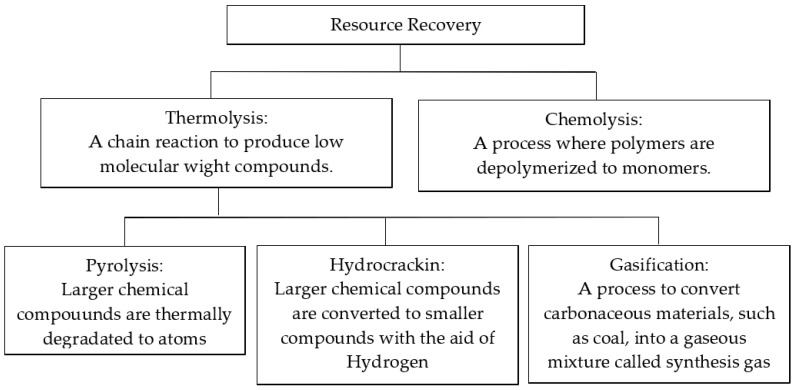
Resource recovery techniques for elimination of plastics.

There are several techniques for disposing plastic wastes properly. One of these methods includes resource recovery which focuses primarily on thermolysis, which is the process where thermoplastics undergo a chain reaction to process low-molecular-weight compounds [69,71]. Thermolysis can be further broken down into three more techniques known as pyrolysis, hydrocracking, and gasification. Figure 7 shows the technologies associated with resource recovery and provides a description for each method [69]. Chemo-lysis involves breaking the polymers into monomers in a solvent such as water (hydrolysis), alcohol (alcohol-lysis), or methanol (methano-lysis) [69].

## 6. Summary and Conclusions

It is safe to conclude that from the research on plastic waste generation and consumption, its presence in the ocean, and the transport and fate of MPs, MPs and plastic in general have detrimental effects on living organisms and the environments where they are present. It is proven through research that MPs have direct impacts specifically on humans and aquatic life. Heavy metals and persistent organics may also enhance the toxicity of MPs and alter health outcomes.

For humans, studies confirm that the most common health consequences that are known to occur involve the brain and nervous system, accumulation, inflammation, fetal growth restriction (breastmilk transfer and placental transfer, thrombosis, and toxicity. For aquatic life, specifically fish, it has been determined that MPs impact on fish includes effects on their intestines, gonads, circulatory system, gills, muscle, liver, immune system, and endocrine system. It is imperative to note that the animals that are impacted the most include fish, mollusks, zooplankton, crustaceans, and mammals.

Utilizing mass balance models, it is possible to quantify buoyant microplastics at the surface of oceans across the globe. While doing so, authors considered synthetic polymer production values from 1950 to 2015. By utilizing this research model and a reasonable toxicity concentration of microplastics for the ecosystem, it is possible to estimate the year when MPs in the ocean could potentially become toxic to the food chain and pose an accelerated threat to humanity for different discharge situations. The resulting estimates indicated that for current ocean discharge growth, MPs in oceans could reach toxic levels on a global average within years 2398 and 2456, for emissions leveling off in 2020, MPs in oceans could reach broad toxicity between the years 2408 and 2472, and for MP discharge to stopping in 2020, the toxic limit in oceans could be avoided entirely. Hence, assuming worst-case scenario humanity has approximately a mere 375 years to avoid global ecosystem-level die-offs.

Therefore, it is important to look into remediation strategies that can be implemented in an attempt to tackle the impacts of MPs. Additionally, more emphasis on modeling parameters to determine toxicity needs to be provided, thus, resulting in the ability to determine just how toxic plastics can be and how soon the oceans will become too toxic to sustain life. Lastly, research needs to be refined to develop additional tactics to combat the growing concern of plastics and their effects, specifically on cost-effective and easily demonstratable MP destruction methods.

## Figures and Tables

**Table 1:** Hydrophobicity and density for various types of plastics [14].

Type	Source	Hydrophobic/Hydrophilic	Density
Polyethylene (PE)	Food wrap, shopping bags, detergent bottles, fuel	Hydrophobic	0.857–0.975 g/cm^3^
Polypropylene (PP)	Microbeads in personal care products, plastic bags, straws, bottles	Hydrophobic	0.90–0.92 g/cm^3^
Polystyrene (PS)	Floats, cups	Hydrophobic	0.96–1.05 g/cm^3^
Polyvinyl Chloride (PVC)	Electrical applications, pipes, windows	Hydrophobic	1.38 g/cm^3^
Nylon/Polyamides (PA)	Clothing	Hydrophilic	1.14 g/cm^3^
Cellulose Acetate (CA)	Cigarette filters	Hydrophobic	1.3 g/mL
Polyester (PES)	Clothing	Hydrophobic	1.38 g/cm^3^

**Table 2:** Consumption and inhalation of MPs [43].

Frequency	Daily	Daily	Annually	Annually	Total for Both Routes	Total for Both Routes
Route of exposure	Consumed	Inhaled	Consumed	Inhaled	Daily	Annually
Male children	113	110	41,106	40,225	223	81,331
Male adults	142	170	51,814	61,928	312	113,742
Female children	106	97	38,722	35,338	203	74,060
Female adults	126	132	46,013	48,270	258	94,283
**Total population**	487	509	177,655	185,761	996	363,416

**Table 3:** The effects MPs have on fish based on the body system [44].

Body Part	Effect
Endocrine system	Disruption Estrogenic/antiandrogenic
Immune system	Stress Interference Toxicity
Liver	Toxicity Bioaccumulation
Muscle	Toxicity Bioaccumulation
Olfactory sense	Damage (mediated by immune system)
Gills	Obstruction Adhesion
Circulatory system	Translocation Cardiotoxicity
Intestine	Obstruction Adhesion Damage Low efficiency
Gonads	Growth Degeneration Disruption Toxicity

**Table 4:** Public outreach and communal action plan to reduce MPs within the environment.

Public Outreach and Communal Actions to Reduce MPs Within the Environment
Request protocols to establish recycling objectives
Request to set communal recycling objectives
Improve the collection methods and sorting
Manufacture a mechanism to fine the contamination of recycling stream
Request washing machine manufactures to capture MPs from effluent water
Request for nontoxic plastics
Request methods/rules to diminish plastics that enter water bodies via stormwater
Request mechanisms to lessen littering

## Data Availability

None as no new data generated.
